# Supplementary material for: Existential suffering as a motive for assisted suicide: Difficulties, acceptability, management and roles from the perspectives of Swiss professionals
Source: PLoS One. 2023 Apr 21;18(4):e0284698. doi: 10.1371/journal.pone.0284698 (PMC10121014; doi:10.1371/journal.pone.0284698)
Supplement: S1 Table — (DOCX) [file pone.0284698.s001.docx]

**Supporting information 1**

**S1 Table – Number of participants and their demographics**

**n*=**25

|  | **Palliative care providers (n*)** | **Volunteers from *EXIT* (n*)** | **Primary care providers (n*)** |
| --- | --- | --- | --- |
| **People contacted** | 16 | 6 | 4 |
| **Participants** | 15 | 6 | 4 |
| **Gender**   - **Female** - **Male** | 10  5 | 3  3 | 4  - |
| **Age range** | 43-62 | 49-80 | 52-63 |
| **Years of experience in their domain (range)** | 4-36 | 6-14 | 6-40 |
