# Supplementary material for: Existential suffering as a motive for assisted suicide: Difficulties, acceptability, management and roles from the perspectives of Swiss professionals
Source: PLoS One. 2023 Apr 21;18(4):e0284698. doi: 10.1371/journal.pone.0284698 (PMC10121014; doi:10.1371/journal.pone.0284698)
Supplement: S2 Table — (DOCX) [file pone.0284698.s002.docx]

**Supporting information 2**

**S2 Table – Interview guide**

| TOPIC / THEME | MAIN QUESTIONS | FOLLOW-UP QUESTIONS |
| --- | --- | --- |
| Experience with existential suffering and assisted suicide | Did you already receive requests for assisted suicide having existential suffering as a reason?   - if yes, can you tell me more about it? - in your opinion, why were the reasons of this request “existential”? - what finally happened to this person? | When you have been confronted to this existential suffering, did you explore it?   - if yes, how? - if no, why? |
| Difficulties encountered | Do you feel any difficulties when you face these kinds of requests?   - if yes, which? | Do you think you have the tools to confront them?   - if yes, which? - if no, what would you need? |
| Acceptability of existential suffering as a motive for assisted suicide | Do you think that existential suffering is a justifiable reason for requesting assisted suicide?   - why? |  |
| Management and alternatives | 1. What should we propose to people requesting assisted suicide with a reason of existential suffering?  - which alternatives?  1. What would be your role?  - who else do you imagine having to take care of existential suffering when it’s part of the request for assisted suicide? | Do you think it is your role to explore existential suffering?   - if no, whose role, is it?   If the person doesn’t change his/her mind, how would you ensure that there is nothing else to do? |
