# Supplementary material for: Existential suffering as a motive for assisted suicide: Difficulties, acceptability, management and roles from the perspectives of Swiss professionals
Source: PLoS One. 2023 Apr 21;18(4):e0284698. doi: 10.1371/journal.pone.0284698 (PMC10121014; doi:10.1371/journal.pone.0284698)
Supplement: Attachment — Submitted filename: PLOSOne-RESPONSE TO REVIEWERS_18.12.22.docx [file pone.0284698.s003.docx]

PONE-D-22-01201

Existential suffering as a motive for assisted suicide: difficulties, acceptability, management and roles from the perspectives of Swiss professionals

PLOS ONE

Dear Dr. Gaignard,

Thank you for submitting your manuscript to PLOS ONE. After careful consideration, we feel that **it has merit** but **does not fully meet PLOS ONE’s publication criteria** as it currently stands. Therefore, we invite you to **submit a revised version of the manuscript that addresses the points** raised during the review process.

Please pay particular attention to the **reviewers' comments regarding the clarity of the concepts used in the results and conclusions**, as well as **responding to the other concerns they have raised**.

Please submit your revised manuscript by Sep 18 2022 11:59PM. If you will need more time than this to complete your revisions, please reply to this message or contact the journal office at [plosone@plos.org](mailto:plosone@plos.org). When you're ready to submit your revision, log on to <https://www.editorialmanager.com/pone/> and select the 'Submissions Needing Revision' folder to locate your manuscript file.

Please include the following items when submitting your revised manuscript:

- **A rebuttal letter** that responds to each point raised by the academic editor and reviewer(s). You should upload this letter as a separate file labeled 'Response to Reviewers'.
- **A marked-up copy of your manuscript** that highlights changes made to the original version. You should upload this as a separate file labeled 'Revised Manuscript with Track Changes'.
- **An unmarked version of your revised paper** without tracked changes. You should upload this as a separate file labeled 'Manuscript'.

If you would like to make changes to your financial disclosure, please include your updated statement in your cover letter. Guidelines for resubmitting your figure files are available below the reviewer comments at the end of this letter.

If applicable, we recommend that you deposit your laboratory protocols in [protocols.io](http://protocols.io) to enhance the reproducibility of your results. Protocols.io assigns your protocol its own identifier (DOI) so that it can be cited independently in the future. For instructions see: <https://journals.plos.org/plosone/s/submission-guidelines#loc-laboratory-protocols>. Additionally, PLOS ONE offers an option for publishing peer-reviewed Lab Protocol articles, which describe protocols hosted on [protocols.io](http://protocols.io). Read more information on sharing protocols at <https://plos.org/protocols?utm_medium=editorial-email&utm_source=authorletters&utm_campaign=protocols>.

We look forward to receiving your revised manuscript.

Kind regards,

Jamie Males
Editorial Office
PLOS ONE

Journal requirements:

When submitting your revision, we need you to address these additional requirements.

1. Please ensure that your manuscript meets PLOS ONE's style requirements, including those for file naming. The PLOS ONE style templates can be found at

<https://journals.plos.org/plosone/s/file?id=wjVg/PLOSOne_formatting_sample_main_body.pdf> and

<https://journals.plos.org/plosone/s/file?id=ba62/PLOSOne_formatting_sample_title_authors_affiliations.pdf>".

2. We note that the grant information you provided in the ‘Funding Information’ and ‘Financial Disclosure’ sections do not match. When you resubmit, please ensure that you provide the correct grant numbers for the awards you received for your study in the ‘Funding Information’ section.

3. Your ethics statement should only appear in the Methods section of your manuscript. If your ethics statement is written in any section besides the Methods, please delete it from any other section.

[Note: HTML markup is below. Please do not edit.]

Reviewers' comments:

Reviewer's Responses to Questions

**Comments to the Author**
 **1. Is the manuscript technically sound, and do the data support the conclusions?**

The manuscript must describe a technically sound piece of scientific research with data that supports the conclusions. Experiments must have been conducted rigorously, with appropriate controls, replication, and sample sizes. The conclusions must be drawn appropriately based on the data presented.

Reviewer #1: Yes

Reviewer #2: Yes

Reviewer #3: Partly

**2. Has the statistical analysis been performed appropriately and rigorously?**

Reviewer #1: Yes

Reviewer #2: N/A

Reviewer #3: N/A

**3. Have the authors made all data underlying the findings in their manuscript fully available?**

The [PLOS Data policy](http://www.plosone.org/static/policies.action#sharing) requires authors to make all data underlying the findings described in their manuscript fully available without restriction, with rare exception (please refer to the Data Availability Statement in the manuscript PDF file). The data should be provided as part of the manuscript or its supporting information, or deposited to a public repository. For example, in addition to summary statistics, the data points behind means, medians and variance measures should be available. If there are restrictions on publicly sharing data—e.g. participant privacy or use of data from a third party—those must be specified.

Reviewer #1: Yes

Reviewer #2: Yes

Reviewer #3: Yes

**4. Is the manuscript presented in an intelligible fashion and written in standard English?**

PLOS ONE does not copyedit accepted manuscripts, so the language in submitted articles must be clear, correct, and unambiguous. Any typographical or grammatical errors should be corrected at revision, so please note any specific errors here.

Reviewer #1: Yes

Reviewer #2: Yes

Reviewer #3: Yes

**5. Review Comments to the Author**

Please use the space provided to explain your answers to the questions above. You may also include additional comments for the author, including concerns about dual publication, research ethics, or publication ethics. (Please upload your review as an attachment if it exceeds 20,000 characters)

**Reviewer #1:**

This paper refers to interviews with health professionals and EXIT volunteers conducted in 2016. Results based on these interviews had been published earlier (Gaignard & Hurst BMC Medical Ethics 2019).
The research question is relevant and the analysis of the data was carefully conducted. The methodology and results are comprehensibly described. The discussion is informative and balanced.
Minor points:
I was a little confused regarding the number of participants: The abstract mentions 26 participants. -apparently only 25 interviews were analysed. The exclusion of one participant was apparently due to a technical problem with the recording (l. 128). Does the "second part of the analysis" (l.. 130) mean the dataset of this paper as a whole? Please clarify this in the abstract and in the results section.
The literature included in the discussion could possibly benefit from an update including more recent publications, e.g. Pesut et al. BMC Palliat Care. 2021, Simmons et al. J Pain Symptom Manage 2022

***RESPONSE:*** *We thank the reviewer for these pertinent comments. The following changes have been made:*

- *Indeed, only the stories of 25 participants were analyzed. We have clarified this on page 9 and modified the abstract accordingly.*
- *The fact that the work presented in this article is not the second part of the study but simply a second “focus” has been clarified on page 5 and 9.*
- *The references were updated.*

**Reviewer #2:**

Summary: The manuscript addresses the topic of existential suffering within the context of assisted suicide (AS) in Switzerland. The author engages within recent debates on existential suffering by analysing the ways in which professionals can deal with existential suffering of patients. The first objective of the research is to gain understanding regarding the difficulties professionals can encounter in managing existential suffering. Secondly, the article addresses the question of roles. Thirdly, the article focusses on conflicts that can arise regarding the question whether or not existential suffering is a justifiable reason for AS. The qualitative research relies on twenty-six interviews and uses a grounded theory approach. The scope of the data, as well as the selection of participants of various backgrounds, contributes to the strength of the research and ensures inclusivity of different opinions and attitudes regarding the research topic. The article reads pleasantly and treats a relevant topic.

Methods and theoretical framework: The method of the research is sound and reliable and suits the objectives of the research. The data sample of twenty-six interviews as well as the independent coding of four transcripts guarantees validity. Furthermore, the selected participants seem well-suited for the objectives of the research. Regarding the theoretical framework presented in the background (50-104), a few concepts which seem important to understand and interpret the results are missing. This will be explained in the next section.

Results and conclusion: Overall, the results are presented clearly. However, as mentioned, a few concepts presented in the results are not defined clearly in the introduction. This conceptual unclarity becomes visible in the result section **in which the author relates the code ‘talk about death’ to the category of ‘spiritual support’** (table 3). **What is meant with spirituality as well as with ‘talk about death’ does not become clear**. Depending on what is meant with the concepts, a conversation about death does not necessarily equals a spiritual conversation since a conversation about death could encompass several dimensions. For example, talking about death could refer to (1) the process of dying; (2) the experience of being death; or (3) believes about a possible or non-existent life after death. The first dimension, talking about the process of dying, could also be more of a medical conversation whereas dimension 2 and 3 could be spiritual. **Secondly, and this suggestion relies on the above mentioned conceptual unclarity, a definition of spirituality is lacking**. The author notes in the discussion that existential suffering sometimes is described as spiritual suffering, yet it does not become clear how the author views spirituality herself in relation to existential suffering (359-61). Since the author proposes spiritual support in the conclusion as one of the desired strategies for dealing with existential suffering, a conceptualization of spirituality and spiritual support seems necessary.

Recommendation: Overall, the author performed an excellent task in clarifying the difficulties professionals encounter when dealing with existential suffering in the context of AS. The manuscript offers interesting and relevant insights as well as useful practical guidelines. If the author elaborates on the definition of spirituality and what is meant with spiritual support, the article could meet the criteria for publication.

***RESPONSE:*** *We thank the reviewer for the very good analysis of this part which allowed us to go back into the participants' stories to clarify what they shared about “talk about death” and, more generally, spirituality. Re-reading their stories led to some minor changes in the prevalence of certain subcategories (not of the bigger category "spiritual support") and, more importantly, allowed us to greatly enrich the discussion of this result on pages 24-25 where you will see the major changes.*

**Reviewer #3:**

The paper builds upon an earlier publication from the same qualitative study (Gaignard, Hurst 2019) where the professionals' understandings of existential suffering as part of a wish for assisted suicide have been explored. The main contribution of the present paper are two types of difficulties encountered by these professionals when facing existential suffering in assisted suicide requests and 9 suggested ways of managing existential suffering in such situations. Data are collected in the French speaking part of Switzerland, a country where assisted suicide is legal and there are no legal restrictions in accepting existential suffering as a reason for an assisted suicide request. **This is an important qualitative study, and this second part is worth to be published.**
The manuscript as it is now however as some serious and other minor flaws that should and certainly could be mended.
- Line 60-63: explain from your earlier paper how professionals called the things that you subsume under 'existential suffering'? Was ist part of actor language? Explain in more detail which "different and sometimes compounded, losses of the dimensions of life" have been encountered, as far as this information is needed to understand the present analysis. ***R:*** *Thank you for this comment. We have clarified this on page 3 and hope that this makes it clearer.*
- Discuss and clarify the question, whether existential suffering must be intolerable for the professionals in order to be an acceptable reason. Or did they mean that if existential suffering is among the reasons for requesting assisted suicide it is automatically intolerable? ***R:*** *The criterion of "intolerability" for the patient, which we have clarified on page 3, is part of the conditions set forth by SAMS (Swiss Academy of Medical Sciences). Such suffering is not automatically considered to be intolerable, and we have made this explicit on page 4.*

- Line 85: "our" hospitals are Swiss hospitals? ***R:*** *Yes, change made.*
- Line 91: how does "existential distress" in the EAPC guideline relate to your (or the professionals') definition of existential suffering? ***R:*** *The EAPC does not give a clear definition of what they mean by existential distress but it can be argued that it is close to that of current literature, what we clarified in the text on page 4. However, we do not find it necessary here to return to the definition given by our participants.*

- Line 99ff: Say more clearly that you made a study, which has led to two publications. This paper does not represent the second part of your study but reports from the study with a special focus. It is the second paper from one study! This is unclear for the readers. ***R:*** *Yes, thank you, change made on page 5 (Line 133).*
- When you speak of "roles" (throughout the paper) it is unclear what you mean. I have the impression that you only mean the professionals' roles, not the roles of friends or family members, who however are certainly important in coping with existential suffering nd assisted suicide requests. Be more explicit.

***RESPONSE:*** *Indeed, although the issue of a much broader responsibility than that of the health care professionals is mentioned in the results on page 22, it is no longer mentioned in the discussion. See the changes made in this regard on page 23 and 26.*

- Table 1: Explain why data collected in 2016 are published only now. In the discussion you should also refer to this fact and explain what has changed in the meantime in Switzerland (if any). ***R:*** *This has been specified in Table 1 on page 7. We have also added this point in the discussion on pages 26-27 (in the study limits section) explaining also what has changed since then.*
- Table 1: Be more specific about what kind of grounded theory approach you have used. Constructivist? Charmaz? IPA? Expand on the data analysis methodology. ***R:*** *The model used derives from the grounded theory according to Corbin & Strauss. This has been specified on page 5 as well as in Table 1 on page 7.*
- Line 128-130: You don't need to mention the interview that you could not use for analysis just because it was not recorded. Seems irrelevant. ***R:*** *As this point was not clear to the 1st reviewer, we decided to leave the explanation but we shortened the sentence, see change on page 9.*

- Acceptability section: Interesting that you find physicians differentiate between existential suffering as part of a request and the question whether they should act upon the request. Expand on this distinction, since the participants obviously rely on it. I had however the impression that you lump them together under the category of "acceptability". On which level should acceptability be discussed? If you could bring out more details from your data this would be helpful, otherwise it rather obscures a point that participants themselves took seriously. And the information how many participants considered existential suffering to be "a justifiable reason" for requesting remains unclear, if it not clarified what they meant. On one level (line 179) all reasons are just to be accepted out of respect, but on other levels (should they be assisted in suicide? should I assist?) different considerations are important. You could also expand on which reasons have been given by those who said that existential suffering was NOT a justifiable reason for requesting assisted suicide.

***RESPONSE:*** *We thank the reviewer for this very good comment that allowed us to go back into the participants' stories and realize that the answers to our questions were already there. Namely, their opinions on the acceptability of existential suffering in accessing assisted suicide requests. We are pleased to have clarified this, which helps to highlight the divergent opinions on this subject among professionals.*

*See the completely revised acceptability section on page 13.*

- Management section: This is the main part. It should be expanded in several ways. **The category "respect"** **is not completely clear to me**: Why is not judging the patient respecting the patient? Is remaining neutral not also a way of being indifferent, and indifference would then not belong to respect? **The category "explore the suffering" needs specification**, in order to do justice of the good quote that you give in the table, which is more differentiated as your description of the category. **The category "give meaning" is not clear to me**, should be expanded in more detail. What can be "given" and what will the patient then be capable of doing? Meaning literally cannot be given. **Why is the category "psychological support " not included in the category "working together"?** Interesting is the dilemma they see when psychological support has the meaning of having a disorder or psychiatric disease, and therefore they hesitate to recommend it.

***RESPONSE:*** *Again, we thank the reviewer for his comments that allowed us to review this section thoroughly and make the necessary changes to clarify and discuss interesting results.*

- ***Category “Respect” (changed to “Showing respect”):*** *We specified the sub-category you mentioned as "Not judging patients and being open to their request". After re-reading the stories of these 6 participants, we believe that this label is in its rightful place in the "showing respect" category because it reflects this attitude of openness and respect to adopt in the face of such requests.*
- ***Category “Explore the suffering”:*** *You’re right, it was a bit simplistic and did not reflect the richness of this attitude described by participants. Namely that of entering into contact with their patient to seek to sincerely understand their story and the origin of their suffering. We have therefore renamed this category* ***“Seeking to understand the causes of suffering”*** *and elaborated in the text on page 19 in the hope that this is clearer.*
- ***Category “Give meaning”:*** *You’re right: in fact, there is no meaning to be given to these people in suffering, as it is simply not possible. However, one can offer patients a space, a listening ear, possible insights, allowing them to give some meaning to what they are experiencing, to their history, and to their identity. We thus renamed almost all of these subcategories, the summary of which is below.*

| ***OLD TERMINOLOGY*** | ***NEW*** |
| --- | --- |
| ***Give meaning (the category)*** | ***Helping give meaning*** |
| *Taking care of the identity (sub-category)*   - *Let the patient recount her story* - *Restoring value to the patient* - *Giving back a role to the patient* - *Enhance patients’ resources* - *Taking care of the identity* | *Helping people regain a sense of identity*   - *Letting them recount their story* - *Helping them recognize their value (as human beings)* - *Helping them recognize their role in their family or in society* - *Enhancing their resources* - *Helping them regain a sense of identity* |
| *Give meaning (sub-category)*   - *Give meaning* - *Keep hope until the end* | *Helping people give meaning to what they are experiencing*   - *Helping them give meaning to what they are experiencing* - *Helping them maintain hope* |
| *Suggest activities (sub-category)* | *Suggesting activities* |

*Some clarifications have also been made in the explanation of the result “Helping give meaning” on page 19-20. We then reread each of the quotes to make sure they still fit with what the participants wanted to share. We did not have to make any changes in this regard.*

*These clarifications also made us think of the work of the philosopher and bioethicist Hilde Lindemann whose book we mention in the discussion on page 24.*

- ***Category “Psychological support”:*** *We deliberately chose to distinguish this category from "working together" because psychiatrists and psychotherapists, and what they do, were specifically named by participants, whereas when they referred to “interdisciplinarity”, it was much more vague. As if psychologists and psychiatrists had indeed different expertise than the rest of the therapists/professionals. This has been clarified in the text under the result "psychological support" on page 20.*

*The reluctance to recommend psychological support is indeed an interested point that made us reword the paragraph somewhat on page 21.*

- Line 333: do you mean interprofessionality, not only interdisciplinarity? ***R:*** *interprofessionality, see change on page 23.*
- Line 361 (and also in the description of "spiritual" above: It may be a matter of definition of "spirituality" and "spiritual needs", when, as you say, fewer than expected interviews mentioned them. What was your definition? Are there definitions given by participants or to be derived from the interviews?

***RESPONSE:*** *Regarding this point, please refer to our answer to the 2nd reviewer above. In addition, as mentioned in the "spiritual support" result on page 21, the participants were quite vague on this subject and did not provide us with their definition of spirituality (this was not on our list of questions either). However, we address the definition of spirituality in the discussion on page 25.*

**6. PLOS authors have the option to publish the peer review history of their article (**[**what does this mean?**](https://journals.plos.org/plosone/s/editorial-and-peer-review-process#loc-peer-review-history)**). If published, this will include your full peer review and any attached file**s.


If you choose “no”, your identity will remain anonymous but your review may still be made public.


**Do you want your identity to be public for this peer review?** For information about this choice, including consent withdrawal, please see our [Privacy Policy](https://www.plos.org/privacy-policy).

Reviewer #1: No

Reviewer #2: No

Reviewer #3: **Yes:** Christoph Rehmann-Sutter

[NOTE: If reviewer comments were submitted as an attachment file, they will be attached to this email and accessible via the submission site. Please log into your account, locate the manuscript record, and check for the action link "View Attachments". If this link does not appear, there are no attachment files.]

While revising your submission, please upload your figure files to the Preflight Analysis and Conversion Engine (PACE) digital diagnostic tool, <https://pacev2.apexcovantage.com/>. PACE helps ensure that figures meet PLOS requirements. To use PACE, you must first register as a user. Registration is free. Then, login and navigate to the UPLOAD tab, where you will find detailed instructions on how to use the tool. If you encounter any issues or have any questions when using PACE, please email PLOS at [figures@plos.org](mailto:figures@plos.org). Please note that Supporting Information files do not need this step.
