# Supplementary material for: Existential suffering as a motive for assisted suicide: Difficulties, acceptability, management and roles from the perspectives of Swiss professionals
Source: PLoS One. 2023 Apr 21;18(4):e0284698. doi: 10.1371/journal.pone.0284698 (PMC10121014; doi:10.1371/journal.pone.0284698)
Supplement: Attachment — Submitted filename: PLOSOne_Response to reviewers_March 2023.docx [file pone.0284698.s004.docx]

PLOSOne – Response to reviewers_March 2023

PONE-D-22-01201R1

Existential suffering as a motive for assisted suicide: difficulties, acceptability, management and roles from the perspectives of Swiss professionals

PLOS ONE

Dear Dr. Gaignard,

Thank you for submitting your manuscript to PLOS ONE. After careful consideration, we feel that it has merit but does not fully meet PLOS ONE’s publication criteria as it currently stands. Therefore, we invite you to submit a revised version of the manuscript that addresses the points raised during the review process.

As you will see from the comments below, two of the reviewers are happy with the revisions to your manuscript. **However, one reviewer has a request for clarification**. Please could you revise your manuscript to address this request?

Please submit your revised manuscript by Apr 06 2023 11:59PM. If you will need more time than this to complete your revisions, please reply to this message or contact the journal office at [plosone@plos.org](mailto:plosone@plos.org). When you're ready to submit your revision, log on to <https://www.editorialmanager.com/pone/> and select the 'Submissions Needing Revision' folder to locate your manuscript file.

Please include the following items when submitting your revised manuscript:

- A rebuttal letter that responds to each point raised by the academic editor and reviewer(s). You should upload this letter as a separate file labeled 'Response to Reviewers'.
- A marked-up copy of your manuscript that highlights changes made to the original version. You should upload this as a separate file labeled 'Revised Manuscript with Track Changes'.
- An unmarked version of your revised paper without tracked changes. You should upload this as a separate file labeled 'Manuscript'.

If you would like to make changes to your financial disclosure, please include your updated statement in your cover letter. Guidelines for resubmitting your figure files are available below the reviewer comments at the end of this letter.
(…)

We look forward to receiving your revised manuscript.

Kind regards,

Steve Zimmerman, PhD
Associate Editor, PLOS ONE

Journal Requirements:

Please review your reference list to ensure that it is complete and correct. If you have cited papers that have been retracted, please include the rationale for doing so in the manuscript text, or remove these references and replace them with relevant current references. Any changes to the reference list should be mentioned in the rebuttal letter that accompanies your revised manuscript. If you need to cite a retracted article, indicate the article’s retracted status in the References list and also include a citation and full reference for the retraction notice.

[Note: HTML markup is below. Please do not edit.]

Reviewers' comments:

Reviewer's Responses to Questions

**Comments to the Author**

**1. If the authors have adequately addressed your comments raised in a previous round of review and you feel that this manuscript is now acceptable for publication**, you may indicate that here to bypass the “Comments to the Author” section, enter your conflict of interest statement in the “Confidential to Editor” section, and submit your "Accept" recommendation.

Reviewer #1: All comments have been addressed

Reviewer #2: All comments have been addressed

Reviewer #3: (No Response)

**2. Is the manuscript technically sound, and do the data support the conclusions?**

The manuscript must describe a technically sound piece of scientific research with data that supports the conclusions. Experiments must have been conducted rigorously, with appropriate controls, replication, and sample sizes. The conclusions must be drawn appropriately based on the data presented.

Reviewer #1: Yes

Reviewer #2: Yes

Reviewer #3: Yes

**3. Has the statistical analysis been performed appropriately and rigorously?**

Reviewer #1: N/A

Reviewer #2: N/A

Reviewer #3: Yes

**4. Have the authors made all data underlying the findings in their manuscript fully available?**

The [PLOS Data policy](http://www.plosone.org/static/policies.action#sharing) requires authors to make all data underlying the findings described in their manuscript fully available without restriction, with rare exception (please refer to the Data Availability Statement in the manuscript PDF file). The data should be provided as part of the manuscript or its supporting information, or deposited to a public repository. For example, in addition to summary statistics, the data points behind means, medians and variance measures should be available. If there are restrictions on publicly sharing data—e.g. participant privacy or use of data from a third party—those must be specified.

Reviewer #1: Yes

Reviewer #2: Yes

Reviewer #3: Yes

**5. Is the manuscript presented in an intelligible fashion and written in standard English?**

PLOS ONE does not copyedit accepted manuscripts, so the language in submitted articles must be clear, correct, and unambiguous. Any typographical or grammatical errors should be corrected at revision, so please note any specific errors here.

Reviewer #1: Yes

Reviewer #2: Yes

Reviewer #3: Yes

**6. Review Comments to the Author**

Please use the space provided to explain your answers to the questions above. You may also include additional comments for the author, including concerns about dual publication, research ethics, or publication ethics. (Please upload your review as an attachment if it exceeds 20,000 characters)

**Reviewer #1:** I enjoyed reading the revised paper. By carefully adressing the comments (especially from reviewer 3) the categories have gained further clarity. However, the new subcategory " Showing respect and being open to their request" seems ambiguous to me: **Does "open" mean "willing to consider the request (to hasten death)” - or rather something along the lines of "feeling empathy"?**

Thank you for this comment as indeed this code could be clearer. By rereading the testimonies, participants were more talking about "keeping an open mind" in face of requests for assisted suicide, which is why we renamed this code: “Not judging patients and keeping an open mind”

l. 170: Acceptability of existential suffering as a motive for AS: It has now become clear that and how a distinction was made between the acceptability of the request for AS and the acceptability to grant AS in this case. However, it is now not entirely clear **whether the interviewees meant rather the comprehensibility of the request because of an unbearable suffering (in the sense of a distressing symptom) or the acceptability of the desire (in moral terms)**. In other words: there is a difference between “understanding that someone has a certain wish” and “finding it morally acceptable that someone has a certain wish”.

Thank you for this comment, which indeed reveals an important subtlety. By rereading the testimonies of the participants, we can lean towards the first possibility. That in caring for these people, our participants could really reach a point where they could only see (and understand) the unbearable and refractory suffering of the person and that, in this case, and after having tried other alternatives, they could imagine that assisted suicide becomes a solution.

We have thus specified this point on page 13 (lines 190-191) of the manuscript.

**Reviewer #2:** (No Response)

**Reviewer #3:** Very convincing and carefully written.

**7. PLOS authors have the option to publish the peer review history of their article** (<https://journals.plos.org/plosone/s/editorial-and-peer-review-process#loc-peer-review-history>). If published, this will include your full peer review and any attached files. Do you want your identity to be public for this peer review? (Answer options: Yes, No)
If the authors want to publish it, I am fine with it.

7. PLOS authors have the option to publish the peer review history of their article ([what does this mean?](https://journals.plos.org/plosone/s/editorial-and-peer-review-process#loc-peer-review-history)). If published, this will include your full peer review and any attached files.


If you choose “no”, your identity will remain anonymous but your review may still be made public.


**Do you want your identity to be public for this peer review?** For information about this choice, including consent withdrawal, please see our [Privacy Policy](https://www.plos.org/privacy-policy).

Reviewer #1: No

Reviewer #2: No

Reviewer #3: **Yes:** Christoph Rehmann-Sutter

[NOTE: If reviewer comments were submitted as an attachment file, they will be attached to this email and accessible via the submission site. Please log into your account, locate the manuscript record, and check for the action link "View Attachments". If this link does not appear, there are no attachment files.]

While revising your submission, please upload your figure files to the Preflight Analysis and Conversion Engine (PACE) digital diagnostic tool, <https://pacev2.apexcovantage.com/>. PACE helps ensure that figures meet PLOS requirements. To use PACE, you must first register as a user. Registration is free. Then, login and navigate to the UPLOAD tab, where you will find detailed instructions on how to use the tool. If you encounter any issues or have any questions when using PACE, please email PLOS at [figures@plos.org](mailto:figures@plos.org). Please note that Supporting Information files do not need this step.
